# Noncanonical functions of glucocorticoids: A novel role for glucocorticoids in performing multiple beneficial functions in endometrial stem cells

**DOI:** 10.1038/s41419-021-03893-4

**Published:** 2021-06-12

**Authors:** Se-Ra Park, Seong-Kwan Kim, Soo-Rim Kim, Doojin Kim, Kun-Woo Kim, In-Sun Hong, Hwa-Yong Lee

**Affiliations:** 1 Department of Health Sciences and Technology, GAIHST, Gachon University, Incheon, Republic of Korea; 2 Department of Molecular Medicine, School of Medicine, Gachon University, Incheon, Republic of Korea; 3 Department of Surgery, Gachon University Gil Medical Center, Gachon University School of Medicine, Incheon, Korea; 4 Department of Thoracic and Cardiovascular Surgery, Gachon University Gil Medical Center, Incheon, Republic of Korea; 5 Department of Biomedical Science, Jungwon University, Goesan-gun, Chungcheongbuk-do Republic of Korea

**Keywords:** Adult stem cells, Reproductive disorders

## Abstract

Chronic stress has a negative impact on many fertility-related functions; thus, the recent decline in female fertility seems to be at least partially associated with increased stress. The secretion of glucocorticoids is a typical endocrine response to chronic stress and indirectly reduces uterine receptivity through the hypothalamus-pituitary-gonadal (HPG) axis. However, in addition to its well-known canonical role, the direct effects of chronic stress-induced glucocorticoids on various uterine functions and their underlying molecular mechanisms are complex and have not yet been revealed. Recent studies have found that resident stem cell deficiency is responsible for the limited regenerative potential of the endometrium (the innermost lining of the uterine cavity) during each menstrual cycle, which subsequently increases infertility rates. In this context, we hypothesized that stress-induced glucocorticoids directly damage endometrial stem cells and consequently negatively affect endometrial reconstruction, which is important for uterine receptivity. In addition to its well-known canonical roles, we identified for the first time that cortisol, the most abundant and potent glucocorticoid in humans, directly suppresses the multiple beneficial functions (self-renewal, transdifferentiation, and migratory potential) of human endometrial stem cells through its functional receptor, glucocorticoid receptor (GR). Glucocorticoids inhibit well-known survival signals, such as the PI3K/Akt and FAK/ERK1/2 pathways. More importantly, we also found that immobilization of stress-induced glucocorticoids suppresses the various beneficial functions of tissue resident stem cells in vivo. To the best of our knowledge, this is the first study to investigate the direct effects of glucocorticoids on the regenerative capacity of endometrial stem cells, and the findings will facilitate the development of more promising therapeutic approaches to increase female fertility.

## Introduction

Glucocorticoids play a pivotal role in the response to various stressful challenges by modulating rapid physical responses such as the “fight or flight” response in the case of a threat [1]. Indeed, various stressors, such as anxiety and depression [2], malnutrition [3], and viral infection [4], trigger the secretion of glucocorticoids, which in turn negatively affect fertility in women [1, 5, 6]. At the level of the hypothalamus, stress-induced glucocorticoids indirectly inhibit the synthesis and release of gonadotropins [luteinizing hormone (LH) and follicle-stimulating hormone (FSH)], which in turn reduce estrogen production and subsequent uterine susceptibility. However, it is currently unclear whether this reduced uterine susceptibility is due to the indirect effect of glucocorticoid-induced suppression of estrogen secretion in vivo or the direct inhibitory effect of glucocorticoids on uterine reconstruction. Therefore, in addition to its well-known canonical roles in indirectly regulating various uterine functions through the hypothalamus-pituitary-gonadal axis, particular attention has been recently devoted to the noncanonical functions of glucocorticoid in directly regulating uterine receptivity as a novel regulatory cytokine because its receptor is selectively expressed under specific uterine disease conditions and mediates the outcome of these diseases [7–10]. Consistently, the glucocorticoid receptor (GR) is highly expressed in the stromal compartment of the human endometrium throughout the menstrual cycle [11]. High GR expression within the endometrium provides novel insight into possible direct roles of glucocorticoids in the regulation of endometrial reconstruction, which is primarily supported by tissue-resident stem cells.

Reciprocal communication between the receptive endometrium and the blastocyst is one of the critical aspects of successful implantation and a subsequent pregnancy outcome [12]. The endometrium is one of the most dynamically remodeled tissues; it undergoes remarkable cyclic growth up to approximately 5–7 mm within 7 days during each menstrual cycle [13]. Similar to most other dynamically growing tissues, local endometrial stem cells play an important role in the rapid cyclic reconstruction of the functional endometrial layer [14, 15]. Thus, the consistent activation and recruitment of local endometrial stem cells that can differentiate into specific uterine cell types is necessary to achieve successful implantation and subsequent pregnancy [16]. Indeed, Lucas et al. revealed that a deficiency in proliferative endometrial stem cell populations can significantly restrict the regenerative potential of the endometrial tissue and can subsequently increase the rates of miscarriage and preterm labor [16]. In this context, we hypothesized that stress-induced glucocorticoids suppress the regenerative capacity of the uterine endometrium by suppressing various beneficial functions of endometrial stem cells. However, the noncanonical direct effects of glucocorticoids on endometrial stem cells and the underlying mechanisms involved remain unknown. Consistent with our hypothesis, we found for the first time that glucocorticoids act as potent inhibitory factors for various beneficial endometrial stem cell functions, such as self-renewal, migration, and differentiation capacities in vitro and in vivo. Subsequently, we further investigated the underlying mechanism of the inhibitory effects of glucocorticoids on multiple stem cell functions. Interestingly, in various types of stem cells, glucocorticoids, through the activated receptor GR, inhibit key pro-survival signaling cascades, including the PI3K/Akt and FAK/ERK1/2 pathways, which are involved in diverse cellular functions, such as self-renewal [17, 18], cell recruitment [17, 19], and the capacity for pluripotency/transdifferentiation [17, 20, 21]. Importantly, blocking these signaling cascades with specific inhibitors significantly abolished the glucocorticoid-induced inhibitory effects on various beneficial functions of endometrial stem cells. Taken together, these results suggest that in addition to their previously reported canonical roles in the hypothalamus, glucocorticoids act as a stress-induced factor to directly inhibit the regenerative capacity of the endometrium by suppressing various beneficial functions of endometrial stem cells via the PI3K/Akt and FAK/ERK1/2 signaling cascades. Increased understanding of these stress-related molecular cascades may improve pregnancy outcomes by alleviating stress-associated negative effects on various endometrial stem cell functions and subsequent uterine reconstruction.

## Results

### Glucocorticoids directly suppress various regenerative capacity-related functions of endometrial stem cells in vitro

**Fig. 1:**
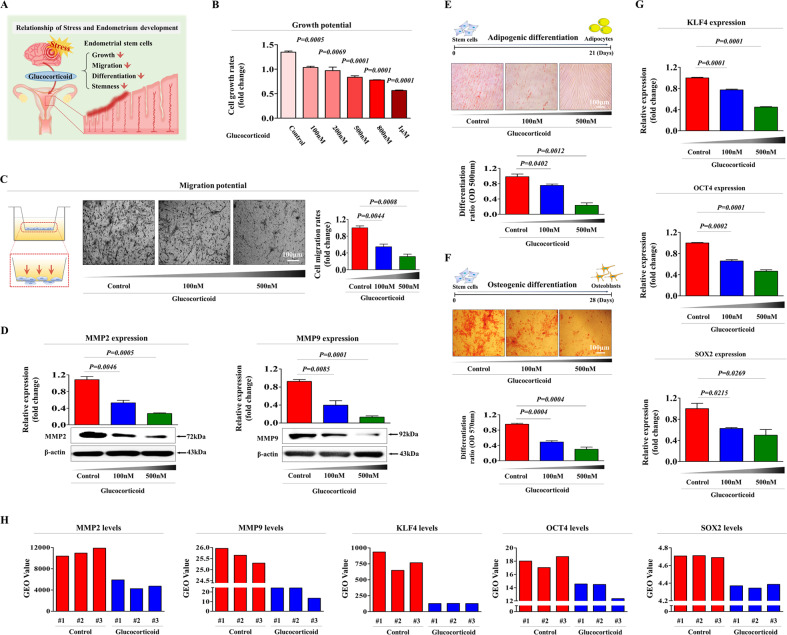
Glucocorticoid treatment significantly inhibits the self-renewal, migration, and transdifferentiation capacity of endometrial stem cells in vitro. We hypothesized that glucocorticoids induced by chronic stress inhibited various regenerative capacity-related functions of endometrial stem cells, including proliferation, migration, pluripotency, and transdifferentiation ability (**A**). The suppression of proliferative capacity by treatment with several concentrations of synthetic glucocorticoid cortisol (100 nM, 200 nM, 500 nM, 800 nM, and 1 µM) was assessed at 72 h by MTT assays. Cell growth rates (%) were determined as the viability of glucocorticoid-treated cells as a percent of the vehicle-treated controls (**B**). Endometrial stem cells were treated with glucocorticoids (100 nM and 500 nM) for 72 h, and the suppressive effects of glucocorticoid treatment on migratory capacity were then assessed using transwell cell invasion assays. Glucocorticoid treatment significantly inhibited the ability of stem cells to migrate across the membrane of transwells (**C**). The expression levels of positive regulators of cell migration (MMP-2 and 9) in response to glucocorticoid treatment were investigated by western blotting (**D**). Endometrial stem cells were cultured for 14 days in adipogenic or osteogenic media with or without glucocorticoid (100 nM and 500 nM) exposure. The suppressive effects of glucocorticoid treatment on adipogenic (**E**) and osteogenic (**F**) differentiation were assessed by oil red O and alizarin red S staining, respectively. The relative quantification of secreted calcium deposition and lipid droplet (LD) formation within differentiated cells was estimated by analyzing the absorbance of the solubilized cells at 500 nm and 570 nm, respectively. The suppressive effects of glucocorticoid treatment on the expression levels of various pluripotency markers (KLF4, OCT4, and SOX2) were measured by real-time PCR (**G**). β-actin was used as the internal control. PPIA was used as a housekeeping gene for real-time PCR analysis. All experiments were performed in triplicates, and the data has been presented as mean ± standard deviation (SD). *P*-value under 0.05 was presented in figures.

Endometrial stem cells were isolated from fresh human endometrial tissue samples and were cultured, as described previously [22] (Supplementary. Fig. 1A). Various cell surface antigens, including CD34, CD44, CD45, CD73, CD105, CD140b, CD146, and susD2, were analyzed by flow cytometry to verify the stemness properties of these cells (Supplementary Fig. 1B). In addition, their ability to transdifferentiate into other cell types were assessed by inducing both adipogenic and osteogenic differentiation in vitro (Supplementary Fig. 1C). A schematic diagram showing the noncanonical stem cell-inhibiting functions of glucocorticoids is illustrated in Fig. 1A. We investigated whether glucocorticoids could restrict endometrial reconstruction by inhibiting the various beneficial functions of endometrial stem cells in vitro as an endogenous stress-associated factor. We first analyzed weather glucocorticoids could induce proliferation of endometrial stem cells and found significantly reduced growth potential in stem cells treated with glucocorticoids compared with nontreated control groups; further, the response was dose-dependent (Fig. 1B). In addition, glucocorticoid treatment also markedly reduced the migratory capacity of endometrial stem cells (Fig. 1C). To further evaluate the inhibitory effect of glucocorticoids on the migratory capacity of endometrial stem cells, the expression levels of MMP-2 and MMP-9, which play important roles in cell migration and invasion by regulating the turnover of the extracellular matrix (ECM), were evaluated using western blotting (Fig. 1D). Importantly, glucocorticoid treatment significantly inhibited the multilineage differentiation capacities of endometrial stem cells toward adipocytes (Fig. 1E) and osteoblasts (Fig. 1F) in a dose-dependent manner. Consistently, the expression levels of several pluripotency-associated transcription factors, KLF4, OCT4, and SOX2, were also substantially reduced by glucocorticoid exposure (Fig. 1G). These in vitro results suggest that glucocorticoids can restrict endometrial reconstruction by inhibiting various regenerative capacity-related functions of endometrial stem cells, such as self-renewal, migratory capacity, transdifferentiation potential and pluripotency.

### Cellular aging-associated phenotypes and metabolic activities of endometrial stem cells are significantly elevated by glucocorticoid treatment in vitro

**Fig. 2:**
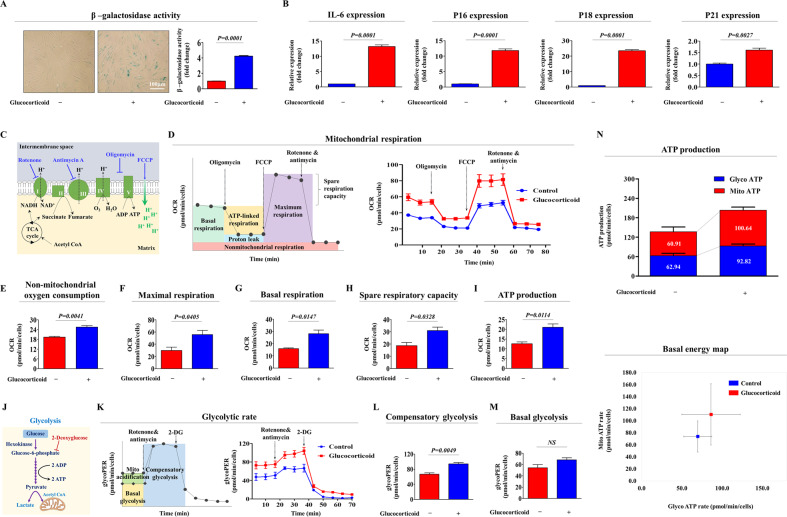
Glucocorticoid exposure significantly increases senescence-associated phenotypes and elevates various metabolic activities in endometrial stem cells. The effects of glucocorticoids on stem cell aging in vitro were investigated by evaluating the enzymatic activities of senescence-associated β-galactosidase (SA-β-Gal) (**A**). The effects of glucocorticoids on the expression levels of the senescence-related markers IL-6, p16, p18, and p21 were evaluated by real-time PCR (**B**). To investigate the effect of glucocorticoid treatment on metabolic activity in endometrial stem cells, mitochondrial oxidative phosphorylation and cytosolic glycolysis were assessed with or without glucocorticoid treatment (**C**). Metabolic profiles of endometrial stem cells in response to glucocorticoid (100 nM) treatment for 72 h were analyzed by measuring the consecutive oxygen consumption rate (OCR) at multiple time steps using a Seahorse XF analyzer. The ATP synthase inhibitor oligomycin (a complex V inhibitor) was used to block mitochondrial respiration. FCCP (an oxidative phosphorylation uncoupler) was added next to disrupt the mitochondrial membrane potential (Δψm). Antimycin A (an inhibitor of complex III of the mitochondrial respiratory chain) and rotenone (an inhibitor of complex I of the mitochondrial respiratory chain) were used to treat cells to completely inhibit mitochondrial oxidative phosphorylation. Mitochondrial respiration rates of endometrial stem cells were largely enhanced in response to glucocorticoid exposure (**D**). Glucocorticoid treatment markedly reduced the nonmitochondrial oxygen consumption rates (**E**), maximal respiration (**F**), basal respiration (**G**), and spare respiratory capacity (**H**) in endometrial stem cells. Overall, ATP synthesis from endometrial stem cells was clearly increased by glucocorticoid treatment (**I**). A schematic diagram of real-time measurements of the glycolytic rate in endometrial stem cells using a Seahorse XF analyzer is shown (**J**). The Seahorse XF glycolytic rate assay uses both OCR and ECAR (extracellular acidification rate) measurements to determine the real-time glycolytic proton efflux rate (glycoPER) of living cells that were cultured in glucose-free media and then subjected to sequential treatment with rotenone, antimycin A, and glucose competitor 2-deoxyglucose (2-DG, a glycolysis inhibitor) in the presence or absence of glucocorticoids (**K**). Compensatory glycolysis is the rate of glycolytic capacity in living cells following the suppression of oxidative phosphorylation and the increase in compensatory energy synthesis through the use of glycolysis to meet changes in energy requirements (**L**). The percentage (%) of PER from glycolytic capacity reveals the contribution of glycolytic activity to total ECAR (**M**). The measured ECAR and OCR values were normalized relative to the total amount of protein per well. The ATP production rates from both glycolytic and mitochondrial pathways were simultaneously measured and quantified in real time using a Seahorse real-time ATP rate assay kit (**N**). All experiments were performed in triplicates, and the data has been presented as mean ± standard deviation (SD). *P*-value under 0.05 was presented in figures.

The most distinct detectable aging-associated phenotype is the presence of senescence-associated beta-galactosidase (SA-β-Gal) activity [23]. Thus, to investigate whether glucocorticoid exposure can induce aging-associated phenotypes, endometrial stem cells were continuously passaged with or without glucocorticoid treatment. Indeed, glucocorticoid treatment significantly increased senescence-related SA-β-Gal activity (Fig. 2A). In addition to SA-β-Gal activity, glucocorticoid exposure markedly increased the expression levels of the following intracellular aging markers: IL-6, p16, p18, and p21 (Fig. 2B). In addition, metabolic changes directly regulate multiple stem cell functions, such as growth potential, migratory capacity, cellular aging, and multilineage differentiation, by controlling energy (ATP) synthesis through glycolysis and/or oxidative phosphorylation [24–26]. Mitochondrial oxidative phosphorylation is regarded as a key indicator of cellular energy-producing potential and of overall cellular health [27]. Therefore, to investigate the effect of glucocorticoids on the energy-producing status of endometrial stem cells, oxidative phosphorylation was measured using an Agilent Seahorse XF analyzer, which allows real-time assessment of metabolic energy utilization by measuring the oxygen consumption rate (OCR) in live cells (Fig. 2C) [28]. To block mitochondrial respiration by inhibiting the electron transport system, stem cells were treated with the ATP synthase inhibitor oligomycin. FCCP (a mitochondrial uncoupler) treatment leads to rapid oxygen consumption without ATP synthesis by decreasing mitochondrial membrane potential (Δψm) [27]. Therefore, we can estimate the maximum rate of mitochondrial respiration that cells can achieve by treating them with FCCP. Interestingly, both mitochondrial respiration (Fig. 2D) and nonmitochondrial oxygen consumption (Fig. 2E) rates of endometrial stem cells were largely enhanced in response to glucocorticoid exposure. Consistently, glucocorticoid treatment also significantly increased the maximal respiration (Fig. 2F), basal respiration (Fig. 2G), and spare respiratory (Fig. 2H) capacities, which are used to determine the substantial amount of extra energy (ATP) that can be synthesized by mitochondria to sustain energy homeostasis in cases of a suddenly increased energy demand [29]. In addition, overall ATP production by oxidative phosphorylation in endometrial stem cells was markedly increased by glucocorticoid treatment (Fig. 2I). We further investigated glycolytic ATP synthesis in endometrial stem cells with or without glucocorticoid treatment by measuring the extracellular acidification rate (ECAR) in real time because lactic acid and protons are generated in cells during the glycolytic process [30, 31]. A schematic diagram of the glycolytic analysis using a Seahorse XF analyzer is described in Fig. 2J. 2-Deoxyglucose (2-DG) was added to inhibit glycolytic activity, which causes basal ECAR levels [32]. Antimycin A (an inhibitor of the electron transport chain at complex III) and rotenone (an inhibitor of the electron transport and proton pump at complex I) were added to inhibit mitochondrial respiration; therefore, further oxygen consumption was completely blocked [33]. Real-time assessment of glycolytic activity revealed that glucocorticoid-treated endometrial stem cells exhibited higher maximal glycolytic capacity than nontreated cells (Fig. 2K). Glucocorticoid treatment also significantly enhanced compensatory glycolytic (Fig. 2L) and basal glycolytic (Fig. 2M) rates. These findings suggested that glucocorticoid treatment markedly increases both anaerobic (cytosolic glycolysis) and aerobic (oxidative phosphorylation) metabolic activities, which in turn can lead to the subsequent acceleration of cellular senescence (aging) in multiple stem cell types [34–36]. Furthermore, we simultaneously measured and quantified the rate of ATP production from both glycolysis and mitochondria in real-time using a Seahorse real-time ATP rate assay kit. Glucocorticoid-treated stem cells exhibited higher ATP production from both glycolytic and mitochondrial pathways (Fig. 2N). The increased metabolic rate may lead to earlier cellular aging because of the accumulation of toxic substances, including superoxide and peroxide hydroxyl radicals, all of which are produced with the increase in metabolic turnover of food to energy [37]. These results suggest that stress-induced glucocorticoids may accelerate the cellular aging process of endometrial stem cells by increasing various metabolic activities.

### The inhibitory effects of glucocorticoids on multiple beneficial functions of endometrial stem cells are mediated through glucocorticoid receptors (GRs)

**Fig. 3:**
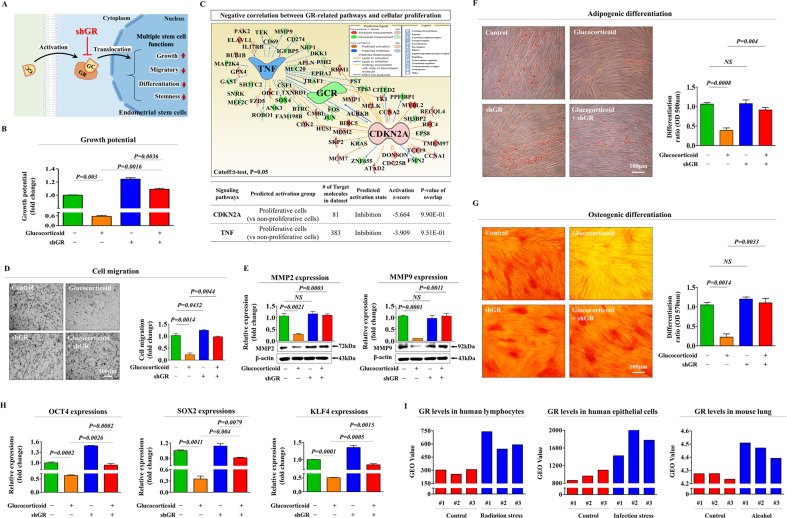
Glucocorticoids exert diverse effects on various regenerative capacity-related functions through their cognate receptors. A schematic diagram regarding the functional role of GR in mediating glucocorticoid-induced effects is shown (**A**). Endometrial stem cells were cultured with 500 nM glucocorticoid alone or were concomitantly transfected with a specific shRNA targeting GR, and subsequent changes in cell proliferation were measured with MTT assays (**B**). Differentially expressed genes in proliferative cells and nonproliferative cells (GSE62564) were investigated by IPA software to assess the activation states of various GR-related pathways/transcription factors (**C**). The suppressive effects of GR depletion on glucocorticoid-induced changes in migratory capacity were also determined by Transwell assays (**D**) and western blotting for MMP-2 and MMP-9 (**E**). The attenuating effects of GR knockdown on the ability of glucocorticoids to suppress adipogenic (**F**) and osteogenic (**G**) differentiation were measured by oil red O staining and alizarin red staining, respectively. The attenuating effects of GR depletion on the expression of the pluripotency-related transcription factors KLF4, OCT4, and SOX2 were determined by real-time PCR (**H**). The GEO database (https://www.ncbi.nlm.nih.gov/geo/) was investigated to analyze the relationship between various physiological conditions and GR expression levels (**I**). β-actin was used as an internal control. PPIA was used as a housekeeping gene for real-time PCR analysis. All experiments were performed in triplicates, and the data has been presented as mean ± standard deviation (SD). *P*-value under 0.05 was presented in figures.

In other cell types (non-stem cells), the effects of glucocorticoids are mediated through glucocorticoid receptors (GRs), which belong to the nuclear receptor superfamily; these group includes receptors for estrogen, mineralocorticoids, retinoic acid, thyroid hormones, and vitamin D [11]. Therefore, to investigate whether glucocorticoids exert their effects through receptor activation in endometrial stem cells, GR was knocked down by stable transfection with an shRNA specifically targeting GR (Supplementary. Fig. 2A, B). A schematic representation of the main hypothesis about the roles of GR as a functional receptor that mediates the inhibitory effects of glucocorticoids is shown in Fig. 3A. Importantly, the glucocorticoid-induced inhibitory effect on self-renewal capacity was markedly abolished by GR depletion (Fig. 3B). To further investigate whether GR-related signaling integrity was associated with self-renewal capacity, we assessed the expression correlation across multiple genes and their signaling networks using ingenuity pathway analysis (IPA). Positive regulators of GR [CDKN2A (Z-score = −5.664, *p*-value = 9.90E-01) and TNF (Z-score = −3.909, *p*-value = 9.51E-01)], which are associated with growth inhibition, were suppressed in highly proliferative cells (Fig. 3C). In addition, glucocorticoid-induced inhibitory effects on migratory capacity (Fig. 3D) and MMP-2/9 expression (Fig. 3E) were significantly attenuated by GR depletion. We also found that glucocorticoid-induced suppressive effects on adipocyte (Fig. 3F) and osteoblast (Fig. 3G) differentiation were markedly abolished by GR depletion. Consistently, the glucocorticoid-induced expression of pluripotency-associated transcription factors, including KLF4, OCT4, and SOX2, was significantly reduced by GR knockdown (Fig. 3H). We then analyzed the gene expression omnibus (GEO) database to further investigate the correlation between GR expression levels and many physiological or pathologic conditions. The levels of GR were also remarkably enhanced in various detrimental conditions (radiation, infection, and alcohol exposure) compared to the levels observed in corresponding controls (Fig. 3I). These results indicate that GR can serve as a functional receptor that mediates the inhibitory effects of glucocorticoids on the various regenerative capacity-related functions of endometrial stem cells.

### Glucocorticoid-induced inhibitory effects on various beneficial functions are mediated through PI3K/Akt and/or FAK/ERK1/2 signaling cascades

**Fig. 4:**
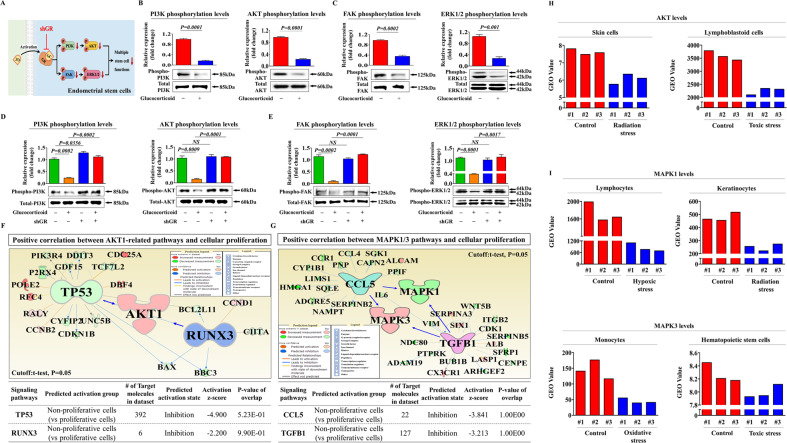
Glucocorticoid-induced inhibitory effects are mediated through the PI3K/Akt and/or FAK/ERK1/2 signaling pathways in endometrial stem cells. A schematic diagram regarding the functional role of the FAK/ERK1/2 and/or PI3K/Akt signaling pathway in mediating glucocorticoid-induced effects is described (**A**). Endometrial stem cells were treated for 10 min with or without glucocorticoids (500 nM). The treated cells were washed with PBS and then lysed, and subsequent changes in protein expression were evaluated by western blotting. The activation states of these signaling molecules (Akt, PI3K, FAK, and ERK1/2) were significantly reduced in cells treated with glucocorticoids (**B**, **C**). Endometrial stem cells were cultured with glucocorticoids (500 nM) alone, or cells were concomitantly treated and transfected with a specific shRNA targeting GR; subsequent changes in the activation states of PI3K, Akt FAK, and ERK1/2 were assessed by western blotting (**D**, **E**). Differentially expressed genes in proliferative cells and nonproliferative cells were investigated using IPA software to determine the activation states (either inhibited or activated) of the Akt1 (GSE116436) (**F**) or MAPK1/3 (ERK1/3) (GSE2034) (**G**) -related pathway/transcription factors. The GEO database was analyzed to further investigate the relationship between the levels of Akt (**H**) or MAPK1/3 (**I**) and various deleterious conditions, such as radiation stress, toxic exposure, or hypoxic stress. β-actin was used as the internal control. All experiments were performed in triplicates, and the data has been presented as mean ± standard deviation (SD). *P*-value under 0.05 was presented in figures.

**Fig. 5:**
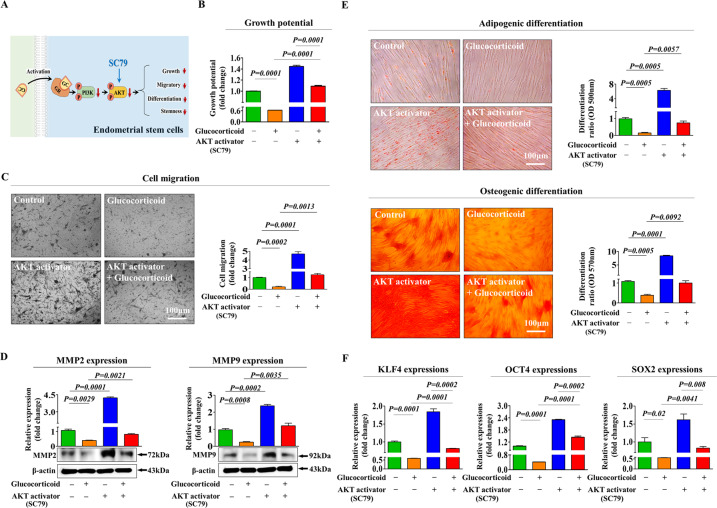
Activation of the Akt signaling pathway attenuates glucocorticoid-induced inhibition of various stem cell functions. A schematic diagram regarding the functional role of the PI3K/Akt signaling pathway in mediating glucocorticoid-induced effects is shown (**A**). Endometrial stem cells were pretreated with the Akt activator SC79 (10 µM) for 1 h prior to treatment with glucocorticoids (500 nM) for 48 h, and the subsequent glucocorticoid-induced changes in cell proliferation were assessed by MTT assays. The percentage (%) of proliferating stem cells was calculated relative to the number observed in the vehicle control (**B**). The attenuating effects of Akt activation on glucocorticoid-induced changes in migratory capacity were analyzed by Transwell assays (**C**) and western blotting for MMP-2 and MMP-9 (**D**), respectively. Endometrial stem cells were pretreated with the Akt activator SC79 (10 µM) for 1 h prior to an additional 48 h treatment with 500 nM glucocorticoids, and subsequent changes in adipocytes and osteoblast differentiation were measured by oil red O and alizarin red staining, respectively. The relative quantification of secreted calcium deposition and lipid droplet (LD) formation within differentiated cells was estimated by analyzing the absorbance of the solubilized cells at 500 nm and 570 nm, respectively (**E**). The attenuating effects of the Akt activator SC79 (10 µM) on glucocorticoid-induced changes in the expression levels of the pluripotency-related transcription factors KLF4, OCT4, and SOX2 were determined by real-time PCR (**F**). β-actin was used as an internal control. PPIA was used as a housekeeping gene for real-time PCR analysis. All experiments were performed in triplicates, and the data has been presented as mean ± standard deviation (SD). *P*-value under 0.05 was presented in figures.

**Fig. 6:**
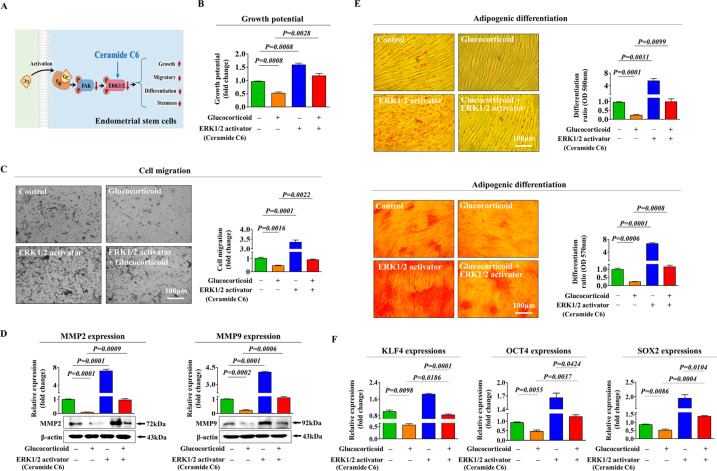
Activation of the ERK1/2 signaling pathway attenuates glucocorticoid-induced inhibitory effects on various stem cell functions. A schematic diagram regarding the functional role of the FAK/ERK1/2 signaling pathway in mediating glucocorticoid-induced effects is shown (**A**). Endometrial stem cells were pretreated with the ERK1/2 activator ceramide C6 (10 µM) for 1 h prior to treatment with 500 nM glucocorticoid for 48 h, and the subsequent glucocorticoid-induced changes in cell proliferation were analyzed by MTT assays. The percentage (%) of proliferating stem cells was calculated relative to the number observed in the vehicle control (**B**). The attenuating effects of ERK1/2 activation on glucocorticoid-induced changes in stem cell migration were analyzed by Transwell assays (**C**) and western blotting for MMP-2 and MMP-9 (**D**), respectively. Endometrial stem cells were pretreated with the ERK1/2 activator ceramide C6 (10 µM) for 1 h prior to an additional 48 h treatment with 500 nM glucocorticoids, and subsequent glucocorticoid-induced changes in adipocyte and osteoblast differentiation were analyzed by oil red O and alizarin red staining, respectively. The relative quantification of secreted calcium deposition and lipid droplet (LD) formation within differentiated cells was estimated by analyzing the absorbance of the solubilized cells at 500 nm and 570 nm, respectively (**E**). The attenuating effects of the ERK1/2 activator ceramide C6 (10 µM) on glucocorticoid-induced expression of the pluripotency-related transcription factors KLF4, OCT4, and SOX2 were measured by real-time PCR (**F**). β-actin was used as an internal control. PPIA was used as a housekeeping gene for real-time PCR analysis. All experiments were performed in triplicates, and the data has been presented as mean ± standard deviation (SD). *P*-value under 0.05 was presented in figures.

To explore the molecular mechanisms underlying the inhibitory effects of glucocorticoids on various regenerative capacity-related functions, we assessed the effects of glucocorticoids on the PI3K/Akt and FAK/ERK1/2 signaling activities, which are involved in the self-renewal [38], migration [39], and differentiation/pluripotency [40] of many types of stem cells. A schematic representation of the main hypothesis that the PI3K/Akt and FAK/ERK1/2 signaling cascades may be involved in glucocorticoid-induced inhibitory effects in stem cells is shown in Fig. 4A. We investigated whether the PI3K/Akt (Fig. 4B) and FAK/ERK1/2 (Fig. 4C) signaling cascades were inhibited by glucocorticoid treatment using western blotting. We then analyzed the effect of GR knockdown on the glucocorticoid-induced inhibitory effects on these signaling pathways. Importantly, glucocorticoid-induced suppressive effects on the PI3K/Akt and FAK/ERK1/2 pathways were markedly attenuated by GR depletion (Fig. 4D, E). To further investigate whether the Akt or ERK (MAPK)1/2-associated signaling activities were positively correlated with the self-renewal ability of various cell types, we assessed the expression correlation across multiple genes and their signaling networks using IPA. Negative regulators of Akt signaling, such as TP53 (Z-score = −4.900, *p*-value= 5.23E-01) and RUNX3 (Z-score = −2.200, *p*-value = 9.90E-01), were suppressed in highly proliferative cells (Fig. 4F). Negative regulators of ERK1/3 (MAPK1/3) signaling, such as CCL5 (Z-score = −3.841, *p*-value = 1.00E00) and TGFβ1 (Z-score = −3.213, *p*-value = 1.00E00), which are associated with growth inhibition, were also suppressed in highly proliferative cells (Fig. 4G). Furthermore, the GEO database indicated that the signaling activities of Akt (Fig. 4H) or MAPK1/3 (Fig. 4I) were remarkably reduced in various degenerative conditions, such as radiation stress, toxic exposure, or hypoxic stress, compared to the levels of corresponding controls. Next, to investigate whether the activation of these signaling pathways abolishes the glucocorticoid-induced inhibition of various beneficial functions, we analyzed the effects of the Akt activator SC79 (Fig. 5A) or the ERK1/2 activator ceramide C6 (Fig. 6A) on self-renewal, migration and multilineage differentiation abilities of cells treated with or without glucocorticoids in vitro. Indeed, the glucocorticoid-induced suppressive effects on self-renewal capacity were significantly attenuated by SC79 (Fig. 5B) or ceramide C6 (Fig. 6B) pretreatment. Consistently, SC79 (Fig. 5C, D) or ceramide C6 (Fig. 6C, D) pretreatment attenuated the glucocorticoid-induced suppressive effects on the migration ability and MMP-2/9 expression of endometrial stem cells. The glucocorticoid-mediated suppressive effects on adipocyte and osteoblast differentiation and the expression of pluripotency-associated transcription factors, such as KLF4, OCT4, and SOX2, were also remarkably abolished by SC79 (Fig. 5E, F) or ceramide C6 (Fig. 6E, F) pretreatment. These results indicate that the PI3K/Akt and/or FAK/ERK1/2 signaling pathways may be involved in glucocorticoid-induced suppression of various beneficial functions in endometrial stem cells.

### Stress-induced glucocorticoids inhibit various regenerative capacity-related functions of endometrial stem cells and subsequent endometrial reconstruction in vivo

**Fig. 7:**
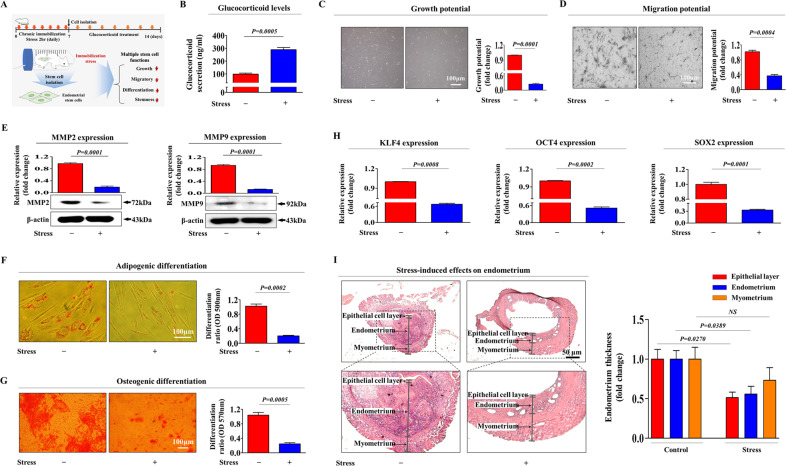
CIS-induced glucocorticoids significantly suppress various regenerative capacity-related functions of endometrial stem cells in vivo. A schematic diagram of the experimental protocol as mentioned in the materials and methods section is described (**A**). The mice were exposed to chronic immobilization stress (CIS, 2 h daily for 7 days), and then endometrial stem cells were isolated from the uterine endometrium. Isolated stem cells were cultured and expanded in vitro with continuous exposure to glucocorticoids (500 nM) to properly mimic physiological conditions of stress-induced glucocorticoid secretion. Compared with the control mice, mice subjected to immobilization-induced chronic stress exhibited a significant increase in glucocorticoid secretion into the peripheral circulation (**B**). The subsequent changes in endometrial stem cell proliferation were assessed by MTT assays. The percentage (%) of proliferating stem cells was calculated relative to the number observed in the vehicle control (**C**). The CIS-induced changes in stem cell migration in vivo were analyzed by transwell assays (**D**) and western blotting with MMP-2 and MMP-9 antibodies (**E**). The effects of CIS-induced glucocorticoids on adipogenic (**F**) and osteogenic (**G**) differentiation in vivo were assessed by oil red O and alizarin red staining, respectively. The relative quantification of secreted calcium deposition and lipid droplet (LD) formation within differentiated cells was estimated by analyzing the absorbance of the solubilized cells at 500 nm and 570 nm, respectively. Real-time PCR results revealed CIS-induced in vivo changes in the expression of several pluripotency-related transcription factors (KLF4, OCT4, and SOX2) (**H**). Uterine endometrial tissue samples from CIS-induced mice were collected and subjected to hematoxylin and eosin (H&E) staining. Histological examination revealed that the endometrial functional layer was significantly decreased by CIS-induced glucocorticoid treatment (**I**). β-actin was used as an internal control. HPRT was used as a housekeeping gene for real-time PCR analysis. All experiments were performed in triplicates, and the data has been presented as mean ± standard deviation (SD). *P*-value under 0.05 was presented in figures.

Our in vitro results indicated that stress-induced glucocorticoids may suppress the regenerative capacity of tissue resident stem cells. In this context, we further investigated whether glucocorticoids inhibit various beneficial functions of tissue resident stem cells and thus negatively affect subsequent endometrial reconstruction in an animal model (Fig. 7A). Importantly, chronic immobilization stress (CIS) resulted in increased glucocorticoid secretion into the peripheral blood flow compared with that of the unstressed control group (Fig. 7B). Consistent with our in vitro data, the in vivo results also indicated that CIS-induced glucocorticoids significantly reduced the self-renewal capacity of local endometrial stem cells (Fig. 7C). In addition, the transwell assay results showed the suppressive effect of CIS-induced glucocorticoids on the migratory capacity of endometrial stem cells in vivo (Fig. 7D). To further investigate the inhibitory effect of CIS-induced glucocorticoids on endometrial stem cell migration, western blotting was used to assess the expression profiles of MMP-2 and MMP-9 (Fig. 7E). CIS-induced glucocorticoids also remarkably reduced the multilineage differentiation capacities of endometrial stem cells toward adipocytes (Fig. 7F) and osteoblasts (Fig. 7G) in vivo. Consistently, the expression of the pluripotency-associated transcription factors KLF4, OCT4, and SOX2 was significantly reduced by CIS-induced glucocorticoids (Fig. 7H). Furthermore, we also evaluated whether CIS-induced glucocorticoids could affect the histological conditions of the uterine endometrium, which was primarily supported by endometrial stem cells. Importantly, histological examination revealed that the functional endometrial layer was significantly decreased by CIS-induced glucocorticoids (Fig. 7I). In addition, we further determined whether CIS-induced glucocorticoids inhibited the beneficial in vivo functions of other types of tissue-resident stem cells, such as adipose tissue-derived stem cells, (Supplementary Fig. 3A). Consistent with the endometrial stem cell results, CIS-induced glucocorticoids significantly decreased the growth potential (Supplementary Fig. 3B), migration (Supplementary Fig. 3C, D), and transdifferentiation capacity (Supplementary Fig. 3E, F) of adipose tissue-derived stem cells. The expression of the pluripotency-associated transcription factors KLF4, OCT4, and SOX2 was also significantly decreased by CIS-induced glucocorticoids in adipose tissue-derived stem cells (Supplementary Fig. 3G). To further evaluate the whether CIS-induced glucocorticoids could affect stem cell populations within uterine endometrial tissue, Mice uterine endometrial tissue samples with or without chronic immobilization stress (CIS) were stained with antibody that is specific for endometrial stem cell marker CD140b. Consistently, our immunostaining results showed that CD140b expression levels were markedly reduced in uterine endometrial tissue samples with chronic immobilization stress (CIS) compared with the corresponding the tissue samples from non-stressed mice (Supplementary Fig. 4). Taken together, these results suggest that CIS-induced glucocorticoids negatively affect subsequent endometrial reconstruction by inhibiting the self-renewal, migratory, and multilineage differentiation capacities of tissue-resident stem cells in vivo.

## Discussion

Tissue resident stem cells, which are more likely located within the basal endometrial layer (stratum basalis), may not be shed during menstruation, and they are primarily responsible for cyclical reconstruction of the uterine endometrium [41]. Indeed, remarkably reduced numbers of active self-renewing resident stem cells within the endometrium were found in approximately 42% of women experiencing recurrent spontaneous abortion, while such a decrease was normally found only in 11% of women who had experienced a successful delivery [16]. Consistently, Lucas et al. also revealed that increased senescence of clonogenic endometrial stem cells can provoke a chronic autoimmune reaction, which might be subsequently associated with a higher prevalence of recurrent pregnancy loss [42]. In this context, intense study of critical regulatory factors responsible for the quality of resident stem cells may provide further insights into the leading causes of endometrium-associated unexplained infertility. Among the various regulatory factors whose functional significance in the endometrium remains undetermined, particular attention has been devoted to the noncanonical functions of glucocorticoids, which act as novel regulatory factors in various uterine functions [7–10].

Increased levels of glucocorticoids in response to stressful challenges result in a diverse range of cellular functions in a variety of cells throughout the body [43, 44], many of which can be associated with reproductive dysfunction by suppressing the hypothalamic-pituitary-gonadal (HPG) axis [6, 45]. At the level of the hypothalamus, glucocorticoids, such as cortisol, suppress the production and secretion of gonadotropin-releasing hormone (GnRH) [46, 47], which in turn decreases estrogen production and subsequently reduces uterine receptivity [48, 49]. In this context, glucocorticoid regulation of uterine functions has historically been considered to have secondary (indirect) effects on the actions of estrogen and progesterone. However, glucocorticoids have recently been investigated as independent direct regulators of various uterine functions. For instance, Shariati et al. found significantly decreased endometrial cell proliferation and a subsequent reduction in uterine receptivity during implantation in animals exposed to glucocorticoids by altering the expression of Muc1, SGK1, ENaC, miRNA 200a, and miRNA 223-3p via the ERK1/2-mTOR signaling pathway [50]. Similarly, Cooper et al. observed a significant increase in the concentration of endometrial uterine NK cells in patients who had received synthetic glucocorticoid prednisolone [51]. Furthermore, by modulating GRα expression, glucocorticoids regulate cyclic changes in endometrial production of prostaglandin F2α (PGF), which is an endometrial receptivity biomarker [10]. Importantly, a recent endocrinological experiment conducted by Nanjappa et al. revealed that the synthetic glucocorticoid dexamethasone significantly inhibited luminal epithelial proliferation in neonatal mouse uteri through both GR-dependent and GR-independent mechanisms [52]. Notably, new challenging questions have arisen regarding the possible direct effect of stress-induced glucocorticoids on various regenerative capacity-related functions of endometrial stem cells and the precise mechanisms underlying the relatively low fertility rate in response to chronic stress. We, therefore, hypothesized that stress-induced glucocorticoids directly reduce the regenerative potential of the uterine endometrium by inhibiting various beneficial functions of tissue-resident stem cells and, as a consequence, may reduce favorable pregnancy outcomes.

Consistent with our hypothesis, glucocorticoid exposure significantly inhibited various beneficial functions of endometrial stem cells, such as the self-renewal (Fig. 1B), migration (Fig. 1C, D), and multilineage differentiation (Fig. 1E, F) capacities, all of which are important for endometrial receptivity and subsequent reproductive outcomes [16, 42, 53–55]. More importantly, in addition to endometrial stem cells, glucocorticoid exposure also significantly reduced various beneficial functions of human adipose tissue-derived stem cells in vivo (Fig. 8A–G), suggesting that stress-induced glucocorticoids may act as a universal inhibitory factor for tissue-resident stem cells that reside in multiple tissue types. In this study, however, uterine endometrial tissue samples from CIS-induced mice and normal mice were isolated form randomly selected mice (not selected according to the estrous cycle). Importantly, the thickness and morphology of endometrium is very dynamically affected by the estrous cycle. Therefore, it is possible that the endometrial changes caused by glucocorticoid exposure are at least partially affected by the changes in steroid hormone during estrous cycle.

Furthermore, the glucocorticoid-induced inhibitory effects on various beneficial functions of endometrial stem cells were markedly attenuated by treatment with the Akt activator SC79 (Fig. 5) or the ERK1/2 activator ceramide C6 (Fig. 6). These results indicate that the PI3K/Akt and/or FAK/ERK1/2 signaling cascades may serve as functional regulators of glucocorticoid-induced inhibitory effects on multiple regenerative capacity-related functions of endometrial stem cells. Consistent with our results, Gupta et al. revealed that synthetic glucocorticoid dexamethasone decreased the phosphorylation levels of Akt ERK1/2 and p38 signaling pathways in human lens epithelial cells without changing proliferation or apoptosis of cells [56]. Kumamaru et al. also found that dexamethasone treatment significantly suppressed ERK1/2 signaling activity in cultured cortical neurons from rats [57].

However, inconsistent with many previous studies, including ours, various synthetic analogs of glucocorticoids are often employed to treat pregnant women at risk of preterm delivery in later pregnancies as treatments for impaired endometrial receptivity by suppression of excessive endometrial inflammation responses [58, 59]. In particular, glucocorticoids can normalize cytokine levels within the endometrium by reducing aberrant populations of immune cells, particularly uterine natural killer (NK) cells [60]. Therefore, while we have confirmed that there are glucocorticoid-induced suppressive effects on various endometrial stem cell functions, it is currently not clear whether glucocorticoids generally improve or adversely affect uterine receptivity and subsequent pregnancy outcomes. Whether glucocorticoids exert the same effects on other cellular components of the uterine endometrium, such as endothelial, stromal, and immune cells, under different conditions also remain unknown and warrants further investigation. Furthermore, these inconsistencies in the results from different studies may also be due to the discrepancies between humans and experimental animal models; animals do not accurately reflect the complex characteristics of human physiology and metabolic activity. Therefore, it may be necessary to develop more appropriate human models for elucidating the long-term effects of stress-induced glucocorticoids in endometrial receptivity.

Taken together, these findings suggest that in addition to their well-known canonical functions, stress-induced glucocorticoids directly decrease various regenerative capacity-related functions of endometrial stem cells through the FAK/ERK1/2 and/or PI3K/Akt signaling pathways. To the best of our knowledge, the present study provides the first evidence that stress-induced glucocorticoids directly affect the regeneration capacity of stem cells. Moreover, our findings may facilitate the development of more effective therapeutic approaches to improve reproductive outcomes for female infertility.

## Materials and methods

### Isolation and culture of human endometrial stem cells

Human endometrial stem cells were obtained from endometrial tissues of uterine fibroid patients with written informed consent from the patients and approval from the Gachon University Institutional Review Board (IRB No: GAIRB2018-134). The endometrial tissue was minced into small pieces, and then the small pieces were digested in DMEM containing 10% FBS and 250 U/ml type I collagenase for 5 h at 37 °C in a rotating shaker. The digestion mixture was then filtered through a 40 µm cell strainer to separate stromal-like stem cells from epithelial gland fragments and undigested tissue. The isolated cells were then cultured in EBM-2 medium (Lonza) with EGM-2 supplements at 37 °C and 5% CO_2_.

### Isolation and culture of mouse uterine tissue-derived stem cells

The isolation of mouse uterine tissue-derived stem cells was approved and conducted in accordance with the Institutional Animal Care and Use Committee (IACUC) (LCDI-2019-0008) of the Lee Gil Ya Cancer and Diabetes Institute of Gachon University. Uterine tissue was minced into small pieces, and then the small pieces were digested in DMEM containing 10% FBS and 250 U/ml type I collagenase for 5 h at 37 °C. The digestion mixture was then filtered through a 40 µm cell strainer. Isolated cells were then cultured in EBM-2 medium (Lonza) with EGM-2 supplements at 37 °C and 5% CO_2_.

### Cell proliferation assay

The MTT assay was used to determine the proliferation-stimulating capacity of synthetic glucocorticoid cortisol (Sigma., Cat. No.: H0888), according to the manufacturer’s protocol (Sigma, Cat. No.: M5655). Cells (1 × 10^4^ cells/well) were seeded in 96-well plates. After 24 h of incubation, the cells were treated with glucocorticoid or vehicle for 72 h. The viable cells were measured at 570 nm using a Versa Max microplate reader.

### In vitro cell migration assay

The stimulating effects of glucocorticoid on the migration potential of endometrial stem cells were evaluated by measuring the number of cells that migrated in response to glucocorticoid treatment divided by the number of spontaneously migrating cells. Cells were plated at 1 × 10^5^ cells/well in 200 μL of culture medium in the upper chambers of permeable Transwell supports (Corning Inc., Corning, NY, USA) to track the migration of cells. The Transwell chambers had 8.0 μm pores in 6.5-mm diameter polycarbonate membranes and used a 24-well plate format. Noninvasive cells on the upper surface of each membrane were removed by scrubbing with laboratory paper. Migrated cells on the lower surface of each membrane were fixed with 4% paraformaldehyde for 5 min and stained with hematoxylin for 15 min. Later, the number of migrated cells was counted in three randomly selected fields of each well under a light microscope at ×50 magnification. The difference in each group is shown as the fold change.

### Protein isolation and western blot analysis

Protein expression levels were determined by western blot analysis, as previously described [61]. Cells were lysed in a buffer containing 50 mM Tris, 5 mM EDTA, 150 mM NaCl, 1 mM DTT, 0.01% NP 40, and 0.2 mM PMSF. The protein concentrations of the total cell lysates were measured by using bovine serum albumin as a standard. Samples containing equal amounts of protein were separated via sodium dodecyl sulfate-polyacrylamide gel electrophoresis (SDS-PAGE) and then transferred onto nitrocellulose membranes (Bio-Rad Laboratories). The membranes were blocked with 5% skim milk in Tris-buffered saline containing Tween-20 at RT. Then, the membranes were incubated with primary antibodies against MMP-2 (Cell Signaling #4022), MMP-9 (Cell Signaling #13667), total PI3K (Cell Signaling #4292), phospho-PI3K (Cell Signaling #4228), total Akt (Cell Signaling #4491), phospho-Akt (Cell Signaling #4060), total-ERK1/2 (Cell Signaling #9012), phospho-ERK1/2 (Cell Signaling #9101), total FAK (Santa Cruz, sc-558), phospho-FAK (Santa Cruz, sc-11765), or β-actin (Abcam, ab189073) overnight at 4 °C and then with HRP-conjugated goat anti-rabbit IgG (BD Pharmingen, 554021) or goat anti-mouse IgG (BD Pharmingen, 554002) secondary antibodies for 60 min at RT. Antibody-bound proteins were detected using enhanced chemiluminescence (ECL) reagents.

### Adipogenic differentiation

Human- or mouse-derived endometrial stem cells were incubated in low-glucose DMEM supplemented with 500 µM methylxanthine, 5 µg/mL insulin, and 10% FBS. Endometrial stem cells were grown for 3 weeks, with medium replacement twice per week. Lipid droplet formation was confirmed by oil red O staining. Relative quantification of lipid droplet formation was performed by measuring absorbance at 500 nm.

### Osteogenic differentiation

Human- or mouse-derived endometrial stem cells were incubated in high-glucose DMEM supplemented with 0.1 µM dexamethasone, 10 mM β-glycerophosphate, 50 µM ascorbate, and 10% FBS. Endometrial stem cells were grown for 3 weeks, with medium replacement twice per week. Differentiated cells were stained with alizarin red S to detect de novo formation of bone matrix. Alizarin red S in samples was quantified by measuring the optical density (OD) of the solution at 570 nm.

### Flow cytometry

FACS analysis and cell sorting were performed using FACS Calibur and FACS Aria machines (Becton Dickinson, Palo Alto, CA), respectively. FACS data were analyzed using FlowJo software (Tree Star, Ashland, OR). Antibodies against the following proteins were used: APC-conjugated CD44 (BD Bioscience, Cat. 559942, dilution 1/40), PE-conjugated CD133 (MACS; Miltenyi Biotech, 130-080-081, dilution 1/40), CD34 (MACS; Miltenyi Biotech, 30-081-002), CD44 (MACS; Miltenyi Biotech, 130-095-180), CD45 (MACS; Miltenyi Biotech, 130-080-201), CD73 (MACS; Miltenyi Biotech, 130-095-182), CD105 (MACS; Miltenyi Biotech, 130-094-941), and CD140b (MACS; Miltenyi Biotech, 130-105-279). The FACS gates were established by staining with an isotype antibody or secondary antibody.

### Real-time PCR

Total RNA from endometrial stem cells was extracted using TRIzol reagent (Invitrogen) according to the manufacturer’s protocol. Real-time PCR was performed using a Rotor-Gene Q (Qiagen). The reaction was subjected to amplification cycles of 95 °C for 20 s, 60 °C for 20 s, and 72 °C for 25 s. The relative mRNA expression of the selected genes was normalized to that of PPIA and quantified using the ΔΔCT method. The sequences of the PCR primers are listed in Table 1.

**Table 1:** Primer sequences for quantitative RT-PCR.

Gene	Gene bank No.	Direction	Primer sequence
Human PPIA	NM_021130	F	TGCCATCGCCAAGGAGTAG
		R	TGCACAGACGGTCACTCAAA
Human KLF4	NM_001314052	F	GAACTGACCAGGCACTACCG
		R	TTCTGGCAGTGTGGGTCATA
Human OCT4	NM_002701	F	AGCCCTCATTTCACCAGGCC
		R	TGGGACTCCTCCGGGTTTTG
Human SOX2	NM_003106	F	AAATGGGAGGGGTGCAAAAGAGGAG
		R	CAGCTGTCATTTGCTGTGGGTGATG
Human GR	NM_000176	F	CAGTGTGCTTGCTCAGGAGA
		R	GTGAGGGTGAAGACGCAGAA
Mouse HPRT	NM_013556	F	GCCTAAGATGAGCGCAAGTTG
		R	TACTAGGCAGATGGCCACAGG
Mouse KLF4	NM_010637	F	GGTGCAGCTTGCAGCAGTAA
		R	AAAGTCTAGGTCCAGGAGGT
Mouse OCT4	NM_013633	F	GCATTCAAACTGAGGCACCA
		R	AGCTTCTTTCCCCATCCCA
Mouse SOX2	NM_011443	F	GAAGCGTGTACTTATCCTTCTTCAT
		R	GAGTGGAAACTTTTGTCCGAGA

### Glucocorticoid receptor (GR) knockdown

Small hairpin RNA targeting GR (shRNA: accession No. NM_000176) and scrambled shRNA (shCTRL) were purchased from Bioneer (Daejeon, South Korea). For efficient shRNA transfection, reverse transfection was performed using Lipofectamine 2000 (Invitrogen, Cat No: 52887) according to the manufacturer’s protocol. We chose the GR shRNA that was most effective at the mRNA level from five shRNAs designed from the target sequence based on qRT-PCR analysis.

### Ingenuity pathway analysis (IPA)

Glucocorticoid receptor-, Akt1-, or MAPK1/3 (ERK1/3)-related gene analyses were performed with IPA version 2.0 software (Ingenuity Systems, Redwood City, CA). Differentially expressed genes (t-test, *P* < 0.005) between nonproliferative cells and proliferative cells were subjected to glucocorticoid receptor- (GSE62564), Akt1- (GSE116436), or MAPK1/3 (ERK1/3) (GSE2034)-related gene analysis. The significance of each factor was measured by Fisher’s exact test (*p*-value), which was used to identify differentially expressed genes from the microarray data that overlapped with genes known to be regulated by a factor. The activation score (Z-score) was used to show the status of predicted factors by comparing the observed differential regulation of genes (“up” or “down”) in the microarray data relative to the literature-derived regulation direction, which can be either activating or inhibiting.

### Analysis of the GEO database

GEO (https://www.ncbi.nlm.nih.gov/geo/) is a freely distributed database repository of high-throughput gene expression data generated by genome hybridization arrays, chip sequencing and DNA microarrays [62, 63]. Researchers provide their experimental results in four categories: experimental designs, samples, platforms, and raw data. Clinical or experimental samples within each dataset are further organized based on various experimental subgroups, such as treatment, physiologic condition, and disease state. These categorized biological data are presented as “GEO profiles”, which include the dataset title, the gene annotation, a chart depicting the expression levels, and the rank for that gene across each sample [64]. Gene expression data were selected from GEO datasets according to multiple parameters, such as tissues, cancers, diseases, genetic modifications, external stimuli, or development. The expression profiles of glucocorticoid receptor (GR), Akt, MAPK1 (ERK1), or MAPK3 (ERK3) in various degenerative conditions were analyzed according to previously established procedures [64].

### Evaluation of the inhibitory effects of CIS-induced glucocorticoid on tissue resident stem cells in animal models

All of the animal experiments were approved and conducted in accordance with the Institutional Animal Care and Use Committee (IACUC) (LCDI-2019-0008) of the Gachon University. The 8-weeks-old female ICR mice were randomly divided into control and immobilization stress (2 h daily for 7 days) exposed groups (each group *n* = 6). The mice were anesthetized and exsanguinated by cardiac puncture, and then stem cells were isolated from uterine and adipose tissues, respectively. For further experiments, isolated stem cells were cultured and expanded in vitro with continuous exposure to glucocorticoid (500 nM) to properly mimic physiological conditions of stress-induced glucocorticoid secretion. Among six mice, three mice in each group were used to make uterine tissue derived stem cells, and the other three mice were used to make paraffin block for H&E staining. The investigators were not blinded to the group allocation and data collection. For further experiments, isolated stem cells from endometrium, adipose tissue, or bone marrow were cultured and expanded in vitro with continuous exposure to glucocorticoid (500 nM) to properly mimic physiological conditions of CIS-induced glucocorticoid exposure in vivo

### Statistical analysis

All in vivo and in vitro data were presented as mean ± S.D. of three independent experimental repeats. All statistical data were analyzed with GraphPad Prism 5.0 (GraphPad Software, San Diego, CA) and evaluated using two-tailed Student’s t-tests. Values of *P* < 0.05 were considered to indicate statistical significance. The variance between the groups was not significant. All the samples were not excluded.

## Supplementary information

Supplementary figures and legends
