# Supplementary material for: Noncanonical functions of glucocorticoids: A novel role for glucocorticoids in performing multiple beneficial functions in endometrial stem cells
Source: Cell Death Dis. 2021 Jun 12;12(6):612. doi: 10.1038/s41419-021-03893-4 (PMC8197759; doi:10.1038/s41419-021-03893-4)
Supplement: Supplementary file 1 — Supplementary figures and legends [file 41419_2021_3893_MOESM1_ESM.docx]

**Supplementary figures and legends**

**
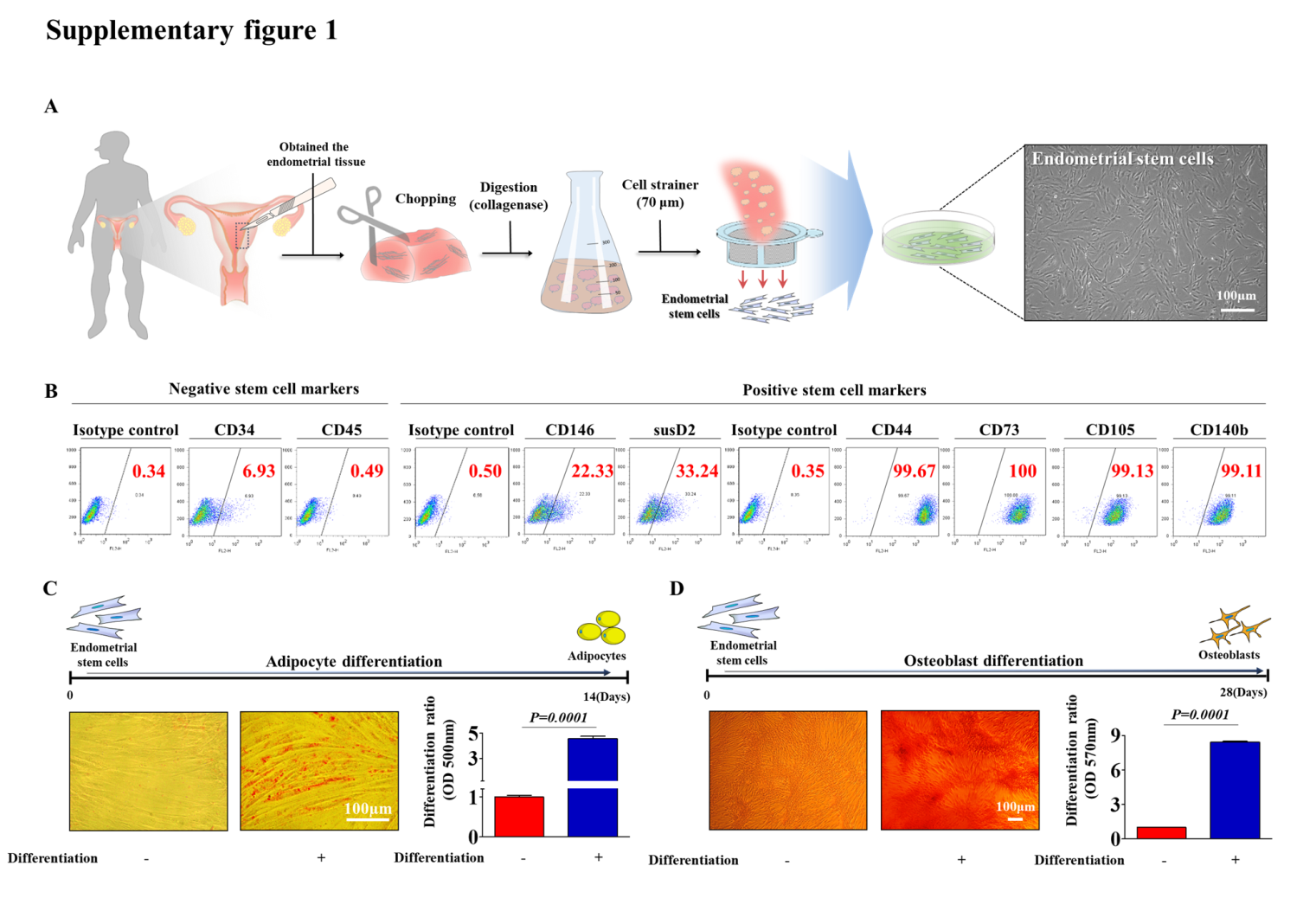
**

**Supplementary Figure 1. Isolation and characterization of human uterine endometrial stem cells.** A schematic diagram showing the isolation procedures for human uterine endometrial stem cells **(A)**. The isolated endometrial stem cells were highly positive for the various known stem cell markers (CD44, CD73, CD105, CD140b, and susD2) and negative for several hematopoietic stem cell markers (CD34 and CD45) **(B)**. The differentiation capacities of these isolated endometrial stem cells into multiple lineages, specifically adipocytes and osteoblasts, were evaluated by oil red O staining and alizarin red staining, respectively. The relative quantification of secreted calcium deposition and lipid droplets (LDs) formation within differentiated cells was estimated by analyzing absorbance of the solubilized cells at 500 nm and 570 nm, respectively. **(C)**. All experiments were performed in triplicates, and the data has been presented as mean ± standard deviation (SD). P-value under 0.05 was presented in figures.

**
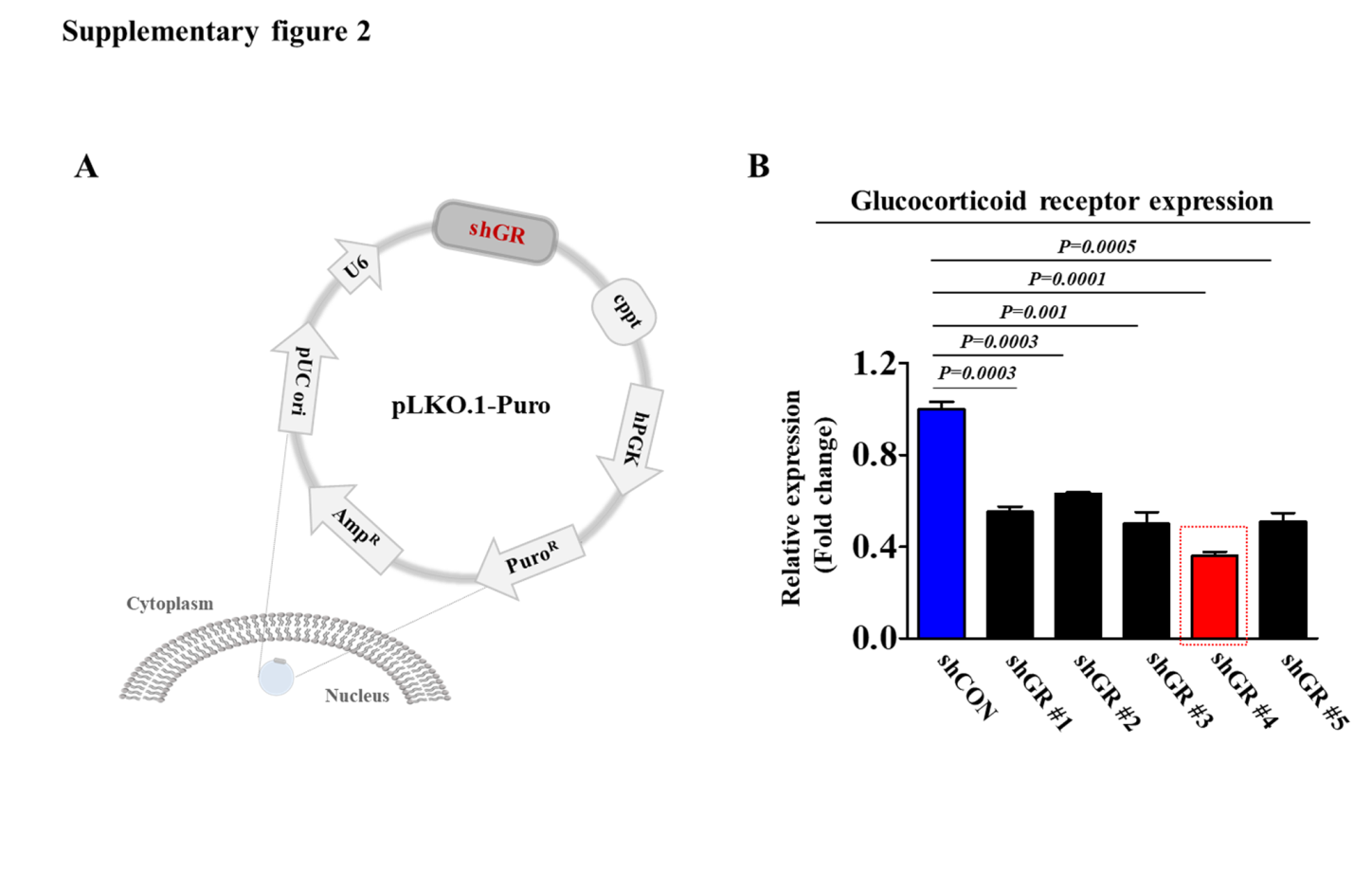
**

**Supplementary Figure 2. Knockdown efficacy of specific shRNAs targeting GR.** Endometrial stem cells were stably transfected with specific shRNA #1, #2, #3, #4, or #5, which targeted GR, or with a nontargeting control shRNA. GR shRNA construct #4, hereafter referred to as GR shRNA, was the most effective for the transfection **(A)**. Successful knockdown of GR expression was verified based on the levels of RNA expression using real-time PCR analysis in endometrial stem cells **(B)**. PPIA was used as a housekeeping gene for real-time PCR analysis. All experiments were performed in triplicates, and the data has been presented as mean ± standard deviation (SD). P-value under 0.05 was presented in figures.

**
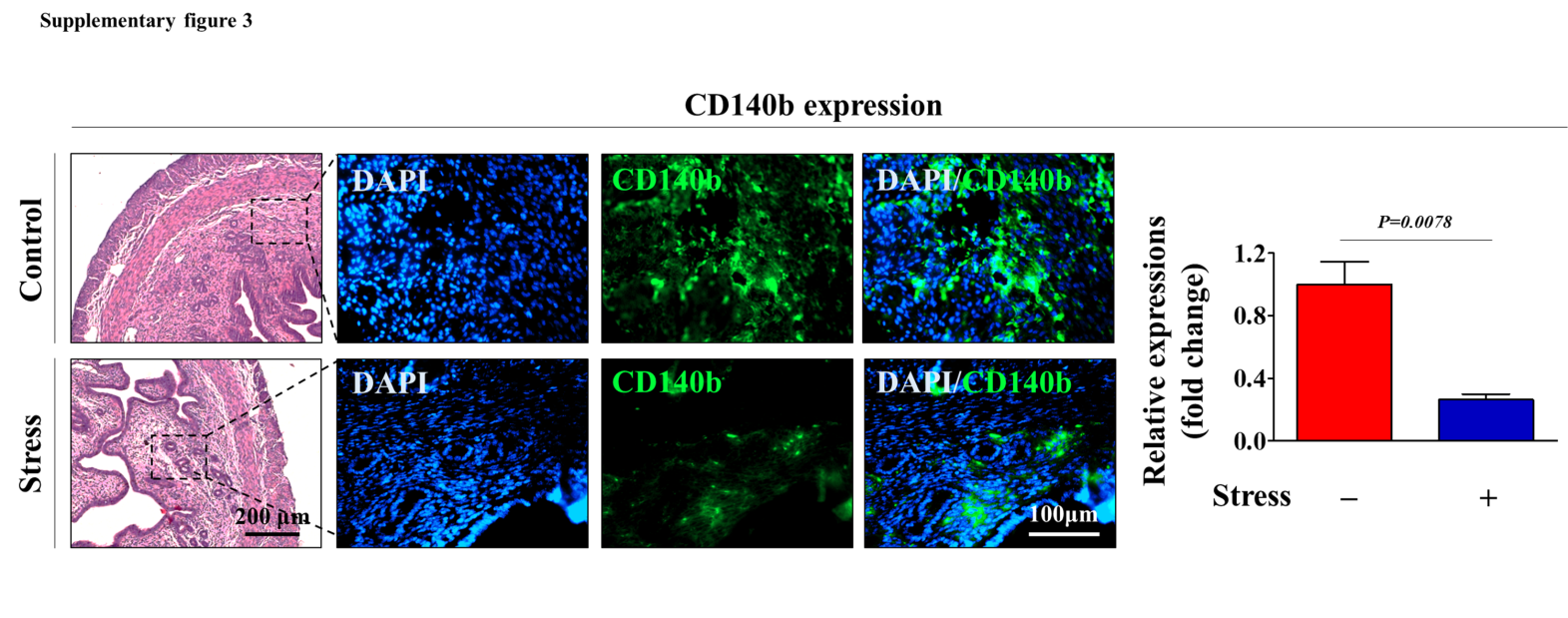
**

**Supplementary Figure 3. The effects of CIS-induced glucocorticoids on endometrial stem cells *in vivo*.** Mice uterine endometrial tissue samples with or without chronic immobilization stress (CIS) were stained with antibody that is specific for endometrial stem cell marker CD140b. DAPI staining was used to label the nuclei within each field. P-value under 0.05 was presented in figures.

**
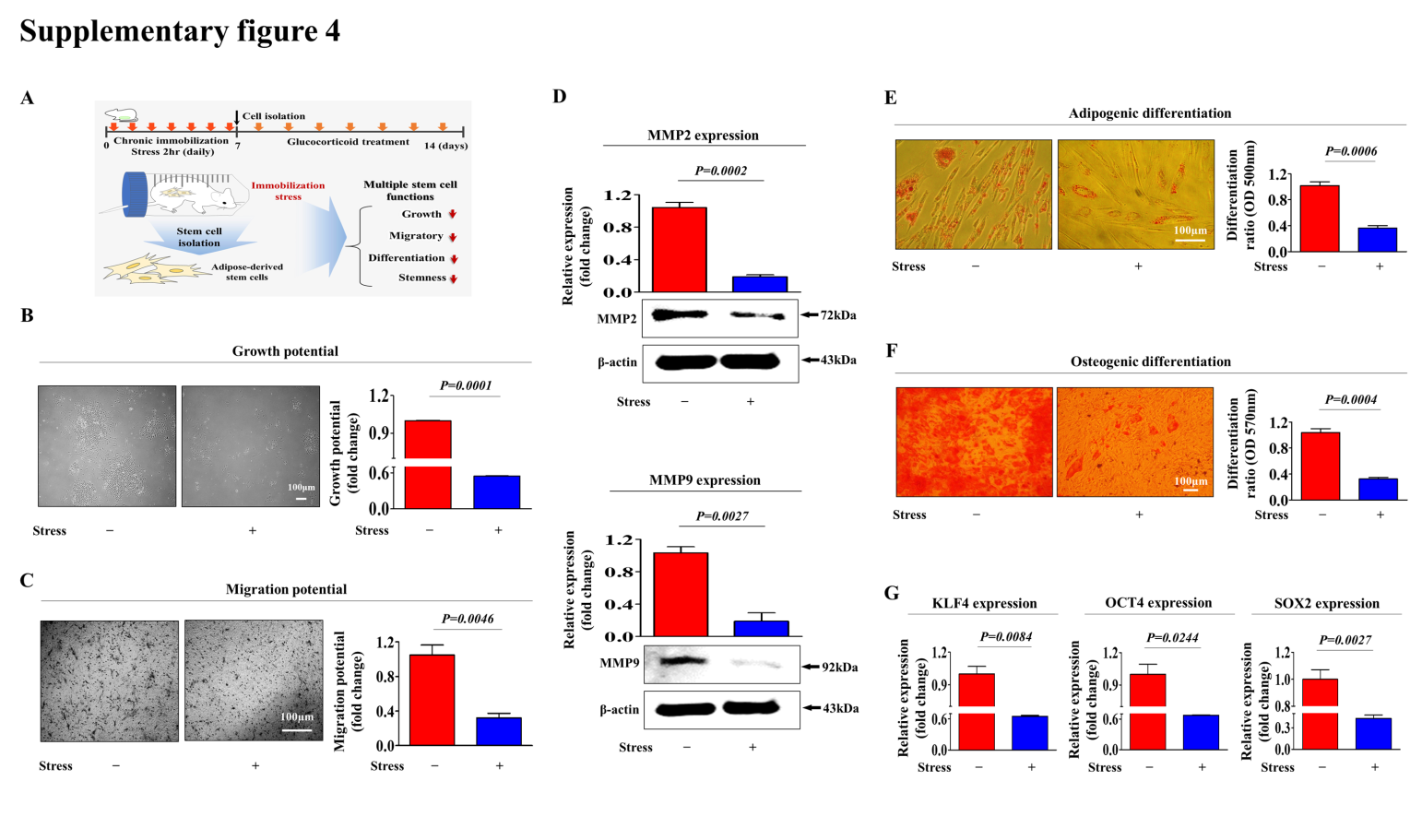
**

**Supplementary Figure 4. CIS-induced glucocorticoid markedly suppresses multiple beneficial functions of other tissue resident stem cells *in vivo*.** A schematic diagram of the experimental protocol as mentioned in the materials and methods section is shown. Similar to what was previously described, the mice were exposed to immobilization stress (2 h daily for 7 days), and then tissue resident stem cells were isolated from adipose tissue. Isolated stem cells were cultured and expanded *in vitro* with continuous exposure to glucocorticoids (500 nM) to properly mimic physiological conditions of stress-induced glucocorticoid secretion. The CIS-induced changes in adipose tissue-derived stem cell proliferation were evaluated by MTT assays. The percentage (%) of proliferating stem cells was calculated relative to the number observed in the vehicle control **(B)**. The CIS-induced changes in stem cell migration *in vivo* were analyzed by transwell assays **(C)** and western blotting with MMP-2 and MMP-9 antibodies **(D)**. The effects of CIS-induced glucocorticoids on adipocyte **(E)** and osteoblast **(F)** differentiation *in vivo* were evaluated by oil red O and alizarin red staining, respectively. The relative quantification of secreted calcium deposition and lipid droplet (LD) formation within differentiated cells was estimated by analyzing the absorbance of the solubilized cells at 500 nm and 570 nm, respectively. Real-time PCR results revealed changes in the expression of several pluripotency-related transcription factors, KLF4, OCT4, and SOX2, after CIS-induced glucocorticoid exposure *in vivo* **(G)**. β-actin was used as an internal control. HPRT was used as a housekeeping gene for real-time PCR analysis. All experiments were performed in triplicates, and the data has been presented as mean ± standard deviation (SD). P-value under 0.05 was presented in figures.
